# Macropore formation in p-type silicon: toward the modeling of morphology

**DOI:** 10.1186/1556-276X-9-585

**Published:** 2014-10-21

**Authors:** Amel Slimani, Aicha Iratni, Hervé Henry, Mathis Plapp, Jean-Noël Chazalviel, François Ozanam, Noureddine Gabouze

**Affiliations:** 1 Unité de recherche matériaux procédés et environnement (UR-MPE), Faculté des sciences de l'ingénieur, Université M'Hamed Bougara, Cité Frantz Fanon, 35000 Boumerdès, Algeria; 2 Physique de la Matière Condensée, École Polytechnique, CNRS, 91128 Palaiseau, France; 3 CRTSE, 2 Bd Frantz Fanon, BP 140, Alger-7 Merveilles 16200, Algeria; 4 Département de physique, Faculté des sciences, Université M'Hamed Bougara, 1, Avenue de l'indépendance, 35000 Boumerdès, Algeria

**Keywords:** Macropore morphology, Porous silicon, Anisotropic etching, Phase-field model

## Abstract

The formation of macropores in silicon during electrochemical etching processes has attracted much interest. Experimental evidences indicate that charge transport in silicon and in the electrolyte should realistically be taken into account in order to be able to describe the macropore morphology. However, up to now, none of the existing models has the requested degree of sophistication to reach such a goal. Therefore, we have undertaken the development of a mathematical model (phase-field model) to describe the motion and shape of the silicon/electrolyte interface during anodic dissolution. It is formulated in terms of the fundamental expression for the electrochemical potential and contains terms which describe the process of silicon dissolution during electrochemical attack in a hydrofluoric acid (HF) solution. It should allow us to explore the influence of the physical parameters on the etching process and to obtain the spatial profiles across the interface of various quantities of interest, such as the hole concentration, the current density, or the electrostatic potential. As a first step, we find that this model correctly describes the space charge region formed at the silicon side of the interface.

## Background

Macropores have first been obtained upon electrochemical dissolution of n-type silicon in hydrofluoric acid (HF)-based electrolytes [1-5], in conditions where the current is limited by the supply of photogenerated holes to the electrochemical interface. For this reason, it has been initially thought that no macropores could be obtained on p-type silicon. However, several experimental groups subsequently proved that such a feeling was erroneous and reported on the growth of macropores on p-Si, either in aqueous or non-aqueous conditions [6-17]. Accounting for these observations became a mandatory task for the theoretical models. A noticeable contribution has been performed by Lehmann and Rönnebeck [18] who tried to extend to the p-Si case the generally admitted model accounting for macropore formation on n-Si, the so-called Lehmann's model [19]. These authors assumed that in the case of p-Si, the electrode is under depletion conditions and that the silicon/electrolyte interface behaves as a Schottky diode. Under these conditions, the hole supply to the interface is limited by the transport across the space charge region (SCR), making the analogy with the n-Si case rather straightforward. In this picture, the holes are focused on the pore tips where the space charge is somewhat thinner due to the 3D configuration of the electric field. Other authors highlight that the coupling of hole transport in the semiconductor and ion transport in the electrolyte could make the semiconductor/electrolyte interface unstable upon dissolution, accounting for the initial formation of pores [20,21]. Since the approach is based on linear stability analysis, it has some intrinsic limitations in describing the pore development and propagation. It has however been refined by Chazalviel et al., who described the practical case where the electrochemical dissolution is under both charge transport and reaction rate control [22]. From this short overview, it appears that taking into account charge transport in the semiconductor and in the electrolyte is needed for accounting for pore formation but that new modeling tools need to be developed in order to account for the pore development and morphology.

The present work is devoted to developing a model which is able to account for macropore formation. We will first recall the experimental results suggesting that such a model should be able to perform a realistic description of charge transport in conditions where silicon is under depletion conditions. Then, we will present first steps toward the development of a phase-field model for macropore formation. The phase-field method has emerged over the last 30 years as a robust method for the solution of moving boundary problems and has been applied to a wide range of phenomena [23-25], including electrochemical growth [26-29]. However, to the best of our knowledge, no phase-field model for silicon dissolution is presently available. The key ingredient for the phase-field approach is to transform the sharp interface into a spatially diffuse one by the use of an order parameter, which is a scalar field *Φ*(*r*) that distinguishes between phases and is hence called phase field. We couple the phase field with the concentrations of two mobile carrier species (holes and ions) and the electric potential in order to construct a minimal model for silicon dissolution. We suppose that the conduction takes place by holes in the semiconductor and by positive ions (cations) in the electrolyte. Both species exist in the whole space, as is the case in any phase-field model; however, their respective concentrations are consistent with the change in conduction mechanism (negligible hole concentration in the electrolyte and negligible ion concentration in the semiconductor).

We present a mathematical analysis as well as numerical simulations of our model and show that it exhibits two new key features that are important for the description of pore formation: at equilibrium, a large space charge region is present on the semiconductor side of the interface, and the application of an external potential yields a nonlinear current-voltage characteristic of Schottky type. Finally, further developments that are needed for a full model of pore formation are discussed.

## Experimental conditions

A (100) electrode was cut from a p-Si single-crystal of 10 Ω cm resistivity. The electrochemical setup consisted of a polystyrene cell and a classical three-electrode arrangement, including a rotating working electrode, a saturated calomel (SCE) reference electrode, and a Pt-wire counter electrode. The electrolyte was prepared from deionized water, ammonium fluoride, and hydrofluoric acid (0.05 M HF, 0.05 M NH_4_F, and 0.9 M NH_4_Cl, i.e., total fluoride concentration *c*_F_ = 0.1 M and pH = 3), which provides slow etching conditions favorable for the elaboration of large-sized macropores [30].

## Macropore morphologies

**Figure 1:**
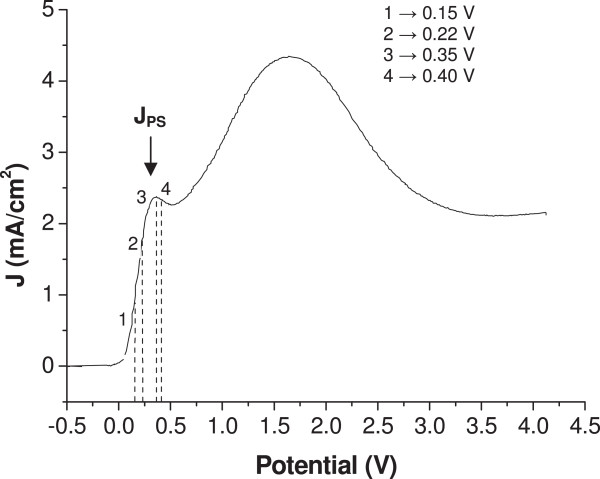
**Voltammogram of a (100) p-Si electrode.** Voltammogram (current density-potential characteristics) of a (100) p-Si electrode in 0.05 M HF, 0.05 M NH_4_F, and 0.9 M NH_4_Cl.

**Figure 2:**
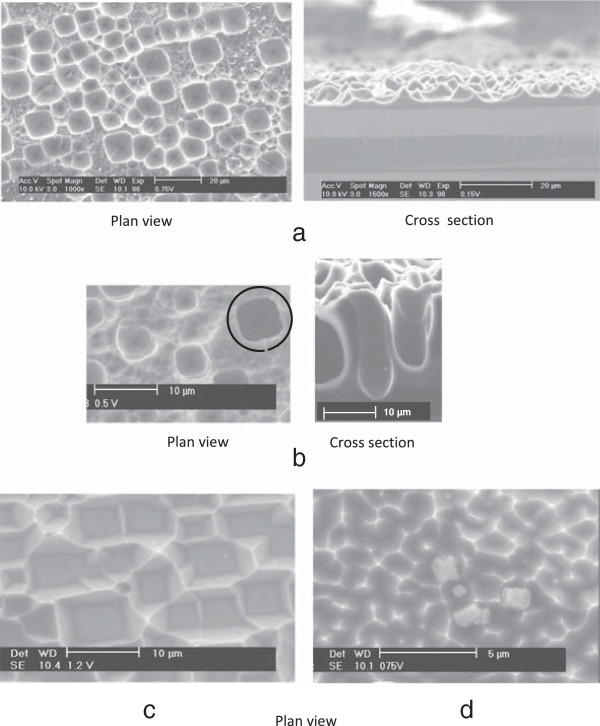
**Pore morphologies, as observed by SEM, obtained for different anodic polarization potentials.** SEM plan view and cross section of the morphologies formed after dissolution of a 10-Ω cm (100) p-Si single-crystal in 0.05 M HF, 0.05 M NH_4_F, and 0.9 M NH_4_Cl for 24 h at **(a)** 0.15, **(b)** 0.22, **(c)** 0.35, and **(d)** 0.40 V.

Figure 1 represents the voltammogram obtained in the present experimental conditions. The rising part of the voltammogram, near 0 V, is known to be associated with the generation of porous silicon. The *I*-*V* characteristics are slightly dependent on the electrode orientation [31]. Figure 2 summarizes the pore morphologies, as observed by SEM, obtained for different anodic polarization potentials. The anodization potentials have been chosen from the voltammogram in Figure 1. At 0.15 V, macrostructures evocative of macropore seeds are found (Figure 2a), but genuine nearly cylindrical macropores grow at longer times only [30]. At 0.22 V, just negative of the potential of the first current peak (*J*_PS_), macropores are formed and invariably exhibit a rounded square shape with rounded-pyramid bottom (Figure 2b). At 0.35 V (the potential corresponding to *J*_PS_), macrostructures are observed (Figure 2c), and at 0.4 V, these macrostructures reduce to thin pyramids (Figure 2d).

The macropore sizes found here are somewhat larger than those found in [14,16,32] for p-Si electrodes of similar doping, which indeed motivated the choice of using a low fluoride concentration in the electrolyte. As for any kind of porous silicon structure, macropores are observed in the rising part of the *I*-*V* characteristics only. This part of the *I*-*V* characteristics has been shown to correspond to a situation where the surface is at least partly hydrogen-covered, in contrast to the situation beyond the current peak where a continuous oxide film covers the surface. From an electrical viewpoint, it means that the interface is not yet under blocking conditions for the charge carriers (unlike in the electropolishing regime) and that the main limitation to carrier transport is likely to be the interfacial Schottky barrier, in agreement with the assumptions of Lehmann and Rönnebeck. However, the macropores more easily form in the region close to the potential corresponding to *J*_PS_, and their shape exhibits a strong dependency on the exact potential value. It suggests that a realistic model for the charge transport at the interface is a mandatory condition for capturing the important features governing the morphology of the macropores. Such is the major goal of the modeling effort presented in the following.

## Phase-field model

### Model formulation

Our goal is to develop a model that is capable of simulating the formation of macropores. This is a highly complex problem, and in the present contribution, we will limit ourselves to the analysis of a stationary planar interface, which can be treated as a one-dimensional problem. The future developments needed to incorporate the anisotropy of the etching are shortly discussed below.

The problem of macropore formation contains multiple length scales. The electrochemical interface exhibits a Helmholtz double-charge layer of atomic width, a diffuse Gouy-Chapman layer on the electrolyte side of the interface, and an extended space charge region in the semiconductor. For the conditions of our experiments, the space charge region typically has a width of 100 to 500 nm, whereas the width of the Gouy-Chapman layer is a few nanometers. Finally, typical pores are a few micrometers in size.

Clearly, it would be highly demanding to resolve all these dramatically different length scales within the same model. Our main goal is to understand the interplay between hole transport in the semiconductor, the geometry of the space charge region, and macropore formation. Therefore, we will set up a simple phenomenological model that works on the scale of the space charge region but neglects all the microscopic details of the Helmholtz and Gouy-Chapman layers.

The electrochemical reaction that we wish to describe reads

(1) $$Si\;+\;4\;HF\;+\;2F^{-}+\;2h^{+}\;\to \;{SiF}_{6}^{2-}\;+\;H_{2}\;+\;2H^{+}$$

Experimentally, ${SiF}_{6}^{2-}$ is the final stable product for silicon dissolution in HF solution. In the semiconductor, the electrical current which drives the reaction is transported by holes, whereas in the electrolyte, both positive and negative ions contribute to the charge transport. In the present work, we will replace the electrolyte by a simpler ‘effective medium’. That is, we assume that there are only two mobile species: holes (h^*+*^) and the mobile cations (H^+^). Indeed, if the negatively charged and neutral species are excluded from reaction (1), it can be seen that the net charge transfer from the semiconductor to the electrolyte can be formally described by these two species. The negative charges are supposed to form a fixed ‘background charge’, which is correct for the semiconductor but is an approximation for the electrolyte.

The interface geometry is described by the phase field *Φ*, which takes the values *Φ =* 1 in the solid Si and *Φ = −*1 in the electrolyte and varies smoothly between these values across a diffuse interface of width *W*_DI_. In the description of phase transitions, the phase field can be interpreted as an order parameter. Since the electrochemical dissolution of silicon is not a phase transition, here the phase field must rather be seen as a smoothed indicator function of the spatial domain occupied by silicon. In this perspective, the detailed form of the interface profile is arbitrary. For a stationary planar interface, the phase field is independent of time and we assume a profile shape

(2) $$\Phi =-\tanh\left(\frac{x}{\sqrt{2}W_{DI}}\right)$$

where *x* is the direction normal to the interface, which is the one-dimensional solution of the equation

(3) $$0=W_{DI}^{2}\nabla^{2}\Phi +\Phi \left(1-\Phi^{2}\right)$$

The electrochemical potentials (the Fermi energies) for the two mobile species holes (h) and ions (i) are given by

(4) $$\xi_{h}=K_{B}T\ln C_{h}+\mathrm{eV}+e\frac{W_{h}}{2}\Phi$$

(5) $$\xi_{i}=K_{B}T\ln C_{i}+\mathrm{eV}+e\frac{W_{i}}{2}\Phi$$

where *K*_B_ is the Boltzmann constant, *T* the temperature, *C* the concentration, *V* the electrostatic potential, and *e* the elementary charge. The first two terms in the above expression represent the contribution of the carrier statistics and the electrostatic energy, respectively. The last terms, *eW*_h_ and *eW*_i_, represent a chemical energy difference between the two media (semiconductor and electrolyte), where *W*_h_ and *W*_i_ are constants with the dimension of an electrostatic potential. These energies can roughly be interpreted as standard levels for each species and determine the concentrations of each type of carrier in the two bulk phases, as will be shown below.

The negative charges are supposed to form a fixed ‘background charge’ *C*_bg_(*Φ*) that is interpolated through the interface as

(6) $$\ln C_{bg}=\frac{1+\Phi }{2}\ln C_{0}^{sc}+\frac{1-\Phi }{2}\ln C_{0}^{el}$$

where $C_{0}^{sc}$ and $C_{0}^{el}$ are the acceptor concentration in the semiconductor and the counterion concentration in the electrolyte, respectively. The form of this interpolation is motivated by the fact that for carrier concentrations that follow Equation 6, the first and last terms in Equations 4 and 5 both vary linearly with *Φ* across the interface.

The holes and the ions together with the background charge determine the local charge density *ρ* = *e*(*C*_h_ + *C*_i_ *− C*_bg_) and thus the electric field or the electrostatic potential *V* via the Poisson equation

(7) $$\nabla \left(\epsilon \nabla V\right)=-\frac{\rho \left(x\right)}{\epsilon_{0}}$$

where ϵ_0_ is the vacuum dielectric permittivity, and *ϵ* is the relative dielectric constant, which is interpolated through the interface by the expression:

(8) $$\epsilon =\left(\frac{1+\Phi }{2}\right)\epsilon_{Si}+\left(\frac{1-\Phi }{2}\right)\epsilon_{el}$$

where *ϵ*_Si_ and *ϵ*_el_ are the relative dielectric constants of silicon and the electrolyte, respectively.

The transport in the semiconductor and in the electrolyte can be described by drift and diffusion terms. The (electric) current density of holes *J*_h_ and cations *J*_i_ is given by:

(9) $$J_{h}=-{µ}_{h}C_{h}\nabla \xi_{h}$$

(10) $$J_{i}=-{µ}_{i}C_{i}\nabla \xi_{i}$$

where *μ*_h,i_ is the mobility of holes and ions, respectively.

The time evolution of the concentrations is given by

(11) $$\partial_{t}C_{h}=\nabla \left(\mu_{h}\;C_{h}\nabla \xi_{h}/e\right)-R\left(\xi_{h},\xi_{i},C_{h},C_{i}\right)$$

(12) $$\partial_{t}C_{i}=\nabla \left(\mu_{i}\;C_{i}\nabla \xi_{i}/e\right)+R\left(\xi_{h},\xi_{i},C_{h},C_{i}\right)$$

The first term is the divergence of the currents, which is the expression for the conservation of species in the absence of the electrochemical reaction. The latter is described by the second term, given by

(13) $$R\left(\xi_{h},\xi_{i},C_{h},C_{i}\right)=\mu_{r}C_{h}C_{i}\left(\xi_{h}-\xi_{i}\right)$$

where *μ*_r_ is a rate constant of dimension of cm^3^/(eV s): the reaction rate is proportional to the product of the concentration of the involved species and to the difference in their electrochemical potentials.

### Interface equilibrium

We now turn to the problem of how to determine suitable model parameters, in particular the two constants *W*_h_ and *W*_i_. To this end, we now analyze the equilibrium properties of the interface. In particular, we wish to determine the concentrations of ions and holes in the two media far from the interface (outside of any space charge region), which we denote by $C_{h}^{Si}$, $C_{i}^{Si}$, $C_{h}^{el}$, and $C_{i}^{el}$, respectively. The following properties of our system are known:

(i) The dopant concentration in the semiconductor $C_{0}^{sc}$. To guarantee the electric neutrality of bulk silicon, the concentrations far from the interface on the silicon side, where *Φ* = 1, must satisfy $C_{h}^{Si}+C_{i}^{Si}=C_{0}^{sc}$.

(ii) The concentration of negative ions $C_{0}^{el}$ in the electrolyte. Again, to guarantee neutrality of the electrolyte, we must have $C_{h}^{el}+C_{i}^{el}=C_{0}^{el}$.

(iii) Since there are no currents at equilibrium, the electrochemical potentials must be constant; in particular, they must be the same in the two bulk media for each species. For example, $\xi_{h}\left(C_{h}^{Si},V_{Si},\Phi =1\right)=\xi_{h}\left(C_{h}^{el},V_{el},\Phi =-1\right)$, where *V*_Si_ and *V*_el_ are the electric potentials in the semiconductor and electrolyte, respectively.

(iv) Since there is also no reaction at equilibrium, the electrochemical potentials for ions and holes must be the same.

The condition *ξ*_h_ = *ξ*_i_, deduced from point (iv), applied to each of the two media, yields

(14) $$\frac{C_{i}^{Si}}{C_{h}^{Si}}=\frac{C_{h}^{el}}{C_{i}^{el}}\equiv R_{C}=\exp\left(\frac{e}{2K_{B}T}\left(W_{h}-W_{i}\right)\right)$$

In order to obtain a model with suitable properties (small amount of ions in the semiconductor, small amount of holes in the electrolyte, thus *R*_*C*_ < <1), we must have *W*_i_ > *W*_h_. In the real system, where typical chemical energies are of the order of 1 eV, the ratio *R*_C_ is vanishingly small. However, in a phase-field model, concentration variations of many orders of magnitude would lead to a very stiff numerical problem, which is impossible to handle. Therefore, we have to choose a value of *R*_C_ that is much larger than in real materials but still small enough to guarantee that the unphysical carriers do not contribute significantly to the currents when an external potential is applied. Once the ratio *R*_C_ is known, the concentrations in each medium can be easily obtained from points (i) and (ii) above. Finally, point (iii) yields the potential difference through the interface at equilibrium (built-in potential),

(15) $$V_{\mathrm{bi}}=V_{Si}-V_{el}=-\frac{\left(W_{h}\;+\;W_{i}\right)}{2}+\frac{K_{B}T}{e}\ln\left(\frac{C_{0}^{el}}{C_{0}^{sc}}\right)$$

This relation shows that the equilibrium potential at the interface is reached when the energy variation of electrostatic origin compensates exactly the energy variation of chemical origin.

Equations 14 and 15 can be used in two ways. Either the constants *W*_i_ and *W*_h_ are known from a microscopic model, and the equilibrium properties of the interface can be evaluated, or if the equilibrium potential difference and the ratio of carrier concentrations are prescribed, corresponding values for the constants *W*_i_ and *W*_h_ can be computed. In both cases, the width of the space charge region (*W*_SCR_) at equilibrium is given by

(16) $$W_{SCR}=\sqrt{\frac{2\epsilon_{0}\epsilon_{Si}V_{\mathrm{bi}}}{eC_{0}^{sc}}}$$

## Simulations

### Numerical method and parameters

The equations developed in the preceding section are discretized on a regular grid of spacing *dx* = 0.5 *W*_DI_ = 5 nm using standard finite-difference formulas. We use a one-dimensional system of 400 grid points (size 2 μm), with the interface located in the center. The concentrations of each species are fixed at the boundaries to the equilibrium values. We choose parameters (listed in Table 1) that are closely related to our experiments. The dielectric constants are those of silicon and water, respectively. The mobility of holes is a typical value from the literature; for the ions (protons), we used a typical diffusion constant of 10^−4^ cm^2^/s together with the Einstein relation to calculate the mobility. The reaction rate constant was set to 10^−4^ cm^3^/(eV s). A semiconductor doped with concentration $C_{0}^{sc}$ is put in contact with an electrolyte of pH = 3 (that is, 10^−3^ mol/l or $C_{0}^{el}$) (Table 1). We choose a built-in potential of *V*_bi_ *=* −0.3 V and a carrier concentration ratio *R*_C_ = exp(−20) ≈ 2 × 10^−9^; this yields the values for *W*_i_ and *W*_h_ listed in Table 1. The Poisson equation was solved at each time step using a successive over-relaxation (SOR) solver. The equations for the carrier concentrations were integrated in time using a simple explicit forward Euler scheme. Small time steps were necessary, especially in the initial stages when the carrier distributions in the interfacial region were far from their final values. Nevertheless, the system eventually converges to an equilibrium or steady-state solution.

**Table 1:** Physical parameters

***W***_**DI**_	***W***_**i**_	***W***_**h**_	***ϵ***_**Si**_	***ϵ***_**el**_	***μ***_**i **_**(H**^**+**^**)**	***μ***_**h**_	$C_{0}^{sc}$	$C_{0}^{el}$
10 nm	0.9599 V	−0.0401 V	11.9	80	4 × 10^−3^ cm^2^ V^−1^ s^−1^	400 cm^2^ V^−1^ s^−1^	10^15^ cm^−3^	6 *×* 10^17^ cm^−3^

### Results

**Figure 3:**
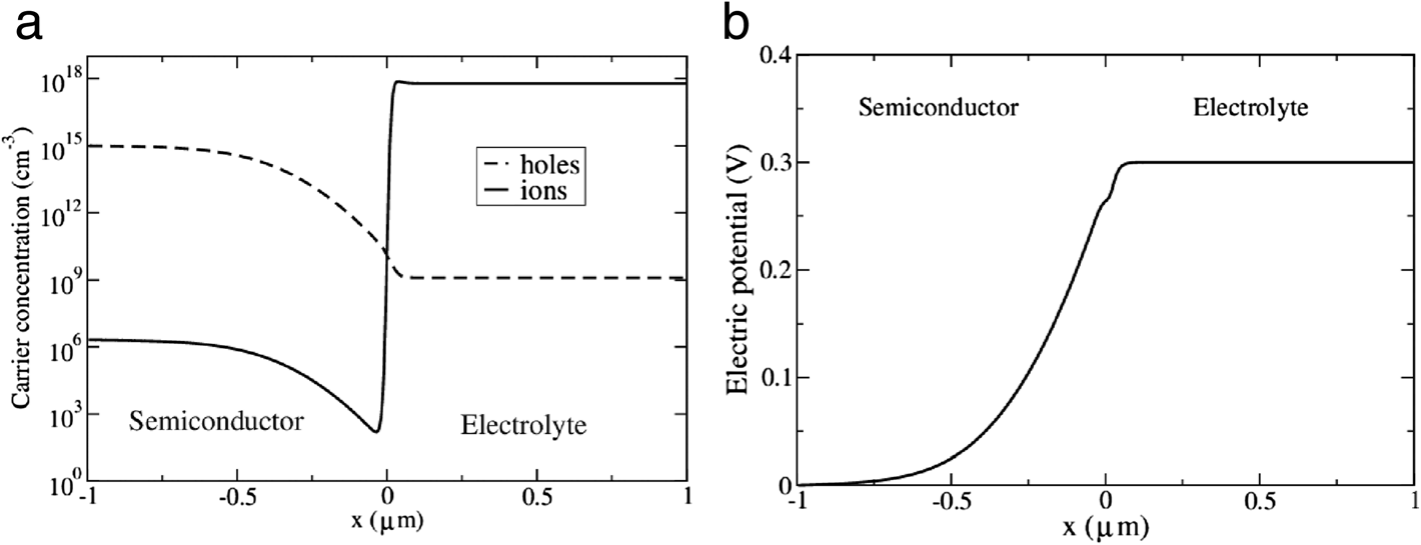
**Equilibrium profiles. (a)** The carrier concentrations: *C*_h_ (hole) and *C*_i_ (cation). **(b)** The electrostatic potential (the flatband potential is 0.3 V).

For the determination of the equilibrium state, we imposed the boundary conditions *V* = 0 on the semiconductor side and *dV*/*dx* = 0 on the electrolyte side (zero current, open circuit conditions). The profile of the hole and cation concentrations through the interface, as well as the electric potential through the interface, is displayed in Figure 3. It can be seen that the model correctly describes the formation of a space charge region that extends considerably into the bulk semiconductor. As a consequence, the electrostatic potential exhibits approximately the characteristic parabolic shape, as shown in Figure 3b. The equilibrium potential difference through the interface is in excellent agreement with the prediction of Equation 15.

**Figure 4:**
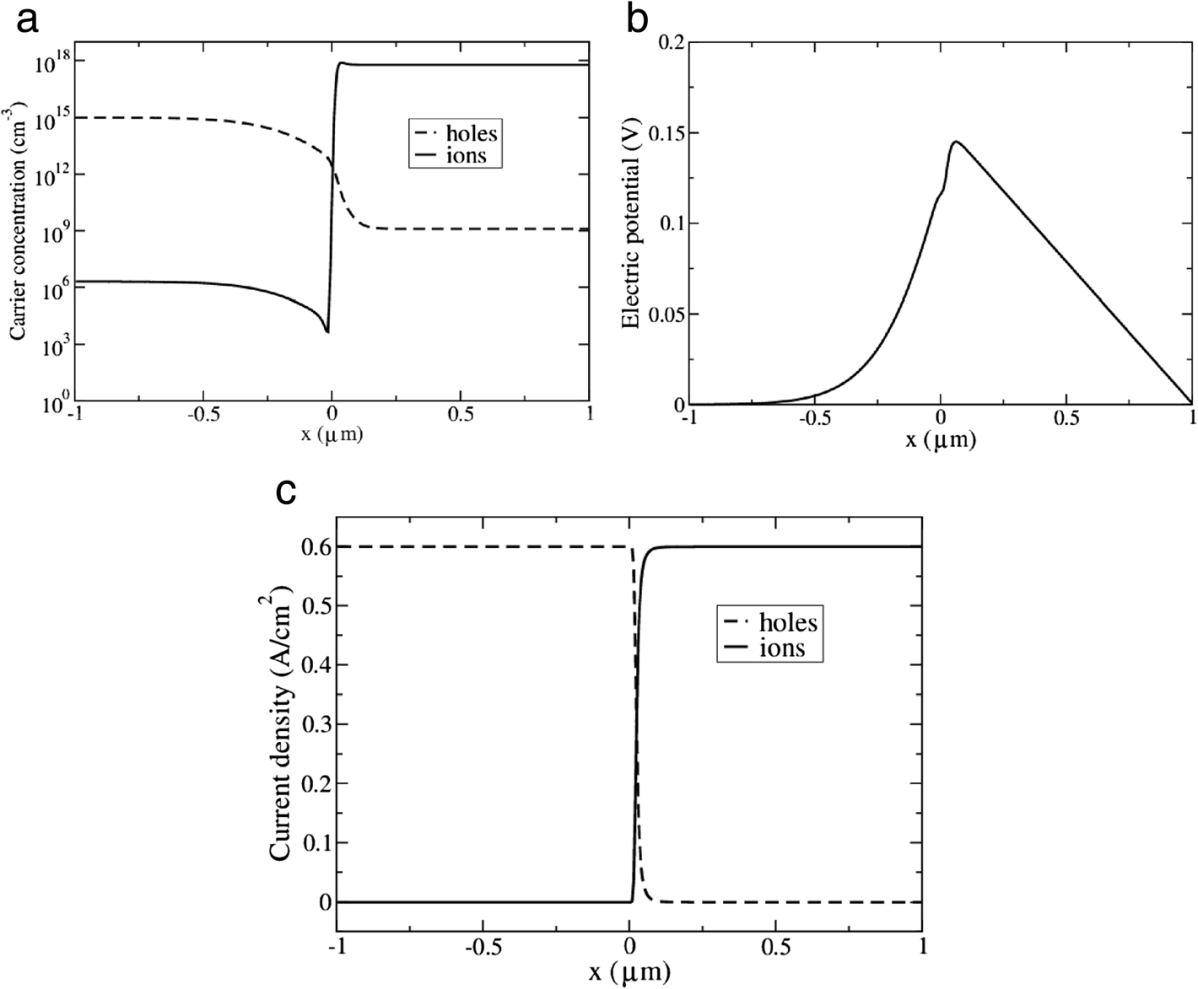
**Profiles under forward bias. (a)** The carrier concentrations: *C*_h_ (hole) and *C*_i_ (cation). **(b)** The electrostatic potential (the flatband potential is 0.3 V). **(c)** The current density: *J*_h_ (hole) and *J*_i_ (cation).

**Figure 5:**
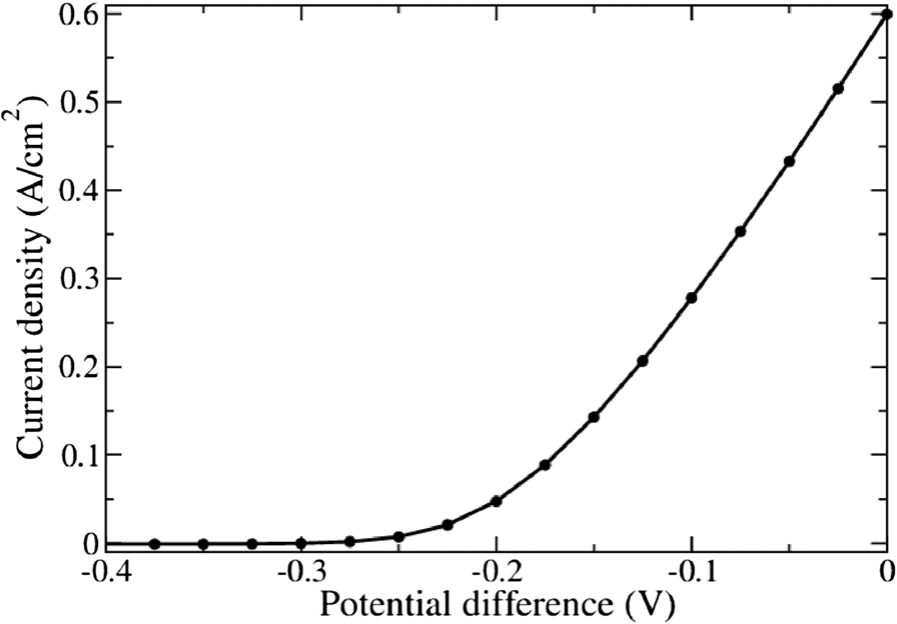
Simulated current density-potential characteristic.

When a potential difference different from *V*_bi_ is applied, a current flows through the system. We stopped our simulations when the relative variations (at different points of the system) of the total current through the system were smaller than 10^−6^. For Δ*V* = 0, the profile of the hole and cation concentrations through the interface is displayed in Figure 4a. The electrostatic potential and the currents of holes and ions are displayed in Figure 4b,c, respectively. It can be seen that the model correctly reproduces the change in conduction mechanism: whereas the hole current is much larger than the ion current inside the silicon, the opposite is true in the electrolyte. The reaction takes place in a narrow region on the electrolyte side of the interface. Furthermore, the *I*-*V* characteristic, shown in Figure 5, has the characteristic nonlinear shape of diode type. This shows that the model, not surprisingly, reproduces the rectifying effect of the space charge region.

## Conclusion

Experimental observations strongly suggest that a proper modeling of the pore morphology should carefully take into account the space charge effects (responsible for the lateral size of the pore/wall structures) at the same time as the chemical dissolution aspects, which represents a real challenge. As a preliminary step toward the modeling of pore formation, we have presented here a phenomenological phase-field model that exhibits a space charge region at a planar interface and correctly reproduces both equilibrium and non-equilibrium properties. In the future, the model will be extended to include interface motion in two dimensions. For this purpose, Equation 3 has to be replaced by an evolution equation for the phase field, with a rate of transformation that is proportional to the electrochemical reaction. Anisotropy can then be included by making the rate constant *μ*_r_ depend on the interface orientation following the standard procedures for phase-field models [33]. While this procedure is straightforward in principle, we expect the numerical calculations (in particular, the solution of the Poisson equation) to become quite heavy. Nevertheless, we believe that, in the long term, this type of model provides a realistic perspective for a modeling of the interplay between the geometry of the space charge region and the macropore formation.

## Competing interests

The authors declare that they have no competing interests.

## Authors’ contributions

AS elaborated the porous silicon, implemented and ran the simulation program, and contributed to the discussion and in the drafting of the manuscript. JNC contributed to the discussion, the drafting and the revision of the manuscript. FO participated in the discussion and contributed in the drafting of the manuscript. MP developed the model and contributed in the drafting of the manuscript. HH contributed to the development of the model and participated in the drafting of the manuscript. AI and NG participated in the discussions. All authors read and approved the final manuscript.
